# Hidden Microbiome of Mining Environments: Implications for Occupational Health, Bioaerosol Exposure, and Antimicrobial Resistance

**DOI:** 10.3390/jox16060194

**Published:** 2026-10-10

**Authors:** Shuaibu Abdullahi Hudu, Emad A. Morad, Ghusun M. Alhazimi, Rashad Qasem Ali Othman, Yahia Abdelgawad Elsayed Elboraei, Abdulgafar Olayiwola Jimoh

**Affiliations:** 1 Center for Health Research, Northern Border University, Arar 91431, Saudi Arabia; 2 Department of Microbiology, Faculty of Medicine, Northern Border University, Arar 91431, Saudi Arabia; 3 Department of Microbiology, Faculty of Medicine, Zagazig University, Zagazig 44519, Al-Sharqia Governorate, Egypt; 4 Department of Bacteriology Lab, Northern Border Regional Laboratory, Northern Borders Cluster, Arar 73211, Saudi Arabia; 5 Department of Pathology, Faculty of Medicine, Northern Border University, Arar 91431, Saudi Arabia; 6 Department of Ear, Nose and Throat Otolaryngology, Faculty of Medicine, Northern Border University, Arar 91431, Saudi Arabia; 7 Department of Pharmacology and Therapeutics, Faculty of Basic Clinical Sciences, College of Health Sciences, Usmanu Danfodiyo University, Sokoto 840232, Sokoto State, Nigeria

**Keywords:** antimicrobial resistance, bioaerosols, environmental microbiology, environmental resistome, environmental surveillance, metagenomics, mining dust

## Abstract

Mining environments are extreme subsurface ecosystems characterized by elevated concentrations of metals and minerals, limited light, and high particulate loads. Occupational health research in mining has traditionally emphasized physical and chemical hazards, whereas biological exposures remain less well-characterized. This narrative review synthesizes evidence on mining-associated microbial communities, bioaerosols, occupational exposure, respiratory health, and antimicrobial resistance (AMR). The literature search covered PubMed/MEDLINE, Scopus, and Web of Science from database inception through August 2026. Evidence from coal, uranium, metal mining, acid mine drainage, and mine dust environments indicates that geochemical conditions and mining activities shape microbial communities. Drilling, blasting, excavation, and ore transport can aerosolize microbial cells, spores, and dust-associated microbial components, creating plausible inhalation pathways. Metal-rich environments may also favor persistence and co-selection of metal- and antibiotic-resistance determinants within environmental microbial communities. Metagenomic sequencing and environmental DNA approaches have expanded detection of microbial diversity, functional genes, and environmental resistomes. However, environmental detection does not establish microbial viability, effective worker exposure, transmission, or disease causation. Future research should integrate standardized environmental and personal microbial exposure measurements with respirable dust and chemical monitoring, longitudinal occupational epidemiology, and functional validation of resistance determinants to determine the actual occupational-health significance of mining-associated microbiomes.

## 1. Introduction

Mining is among the most important industries globally, providing raw materials for energy generation and construction and producing modern technological products. Across the globe, many millions of workers are employed in or around mining operations, often in confined underground spaces with high humidity and temperatures, where air environments can also be substantially contaminated with high levels of particulate matter [1]. Occupational health research in mining has predominantly focused on physical and chemical hazards such as exposure to respirable silica dust, heavy metals, and toxic gases. Such exposures are closely associated with a range of respiratory diseases, including coal workers’ pneumoconiosis, silicosis, and chronic obstructive pulmonary disease, due to long-term inhalation of mineral dust. While researchers have extensively studied the physicochemical hazards of mining environments, their biological aspects remain less explored [2,3]. Mines provide an excellent natural underground ecosystem where extreme geochemical conditions, including high metal concentrations, nutrient starvation, and oxygen fluctuations, impose evolutionary pressure that drives the development of unique microbial communities. Microorganisms colonize diverse niches in mines, including epilithic rock surfaces, mine water systems, ventilation ducts, and dust particles generated during mining activities [4]. In these environments, microbial communities of bacteria, fungi, archaea, and other microorganisms contribute to ecological processes, but their occupational relevance remains incompletely characterized [5]. Bioaerosols, which contain airborne microorganisms and microbial components, are an important but often overlooked component of health risk assessment for workers involved in mining operations. They include bacteria, fungal spores, endotoxins, and viruses [6]. Mining processes such as drilling and blasting can aerosolize these particles and spread them through tunnels and ventilation systems. Inhalation of bioaerosols may contribute to respiratory irritation, allergic responses, inflammation, and, in susceptible individuals and specific exposure settings, respiratory infection [7]. In poorly ventilated areas, airborne microbial concentrations may increase, increasing exposure risks for miners.

So far, researchers have characterized microbial communities and microbial genetic material in mining aerosols and dust [8]. High-throughput sequencing has detected bacterial and fungal taxa, including organisms with recognized pathogenic or opportunistic potential [9]. Mine dust microbiomes are heterogeneous, with complex dynamics that reflect mine-site geology and mining processes [10]. Infectious disease is not the sole health outcome associated with exposure in mining areas, as other outcomes may be affected via proinflammatory or immunologic pathways. Microorganisms activate immune responses through microbial components such as endotoxins and fungal β-glucans, which can induce lung inflammation or hypersensitivity with prolonged exposure [11].

Evidence from other enclosed occupational and healthcare environments also illustrates the value and limitations of environmental microbial surveillance. For example, investigations of SARS-CoV-2 contamination in hospital indoor environments have demonstrated the potential utility of environmental air and surface monitoring for characterizing biological contamination [12]. Such studies provide methodological context for environmental surveillance but are not directly comparable with mining environments because the microbial agents, exposure sources, ventilation characteristics, occupancy patterns, and health endpoints differ. Their relevance to mining microbiome research therefore lies primarily in demonstrating the value of standardized environmental sampling and interpretation alongside direct exposure assessment.

While interest in environmental microbiomes is growing, mining environments are still under-characterized compared with other microbial ecosystems, such as hospital, soil, or urban airborne microbiomes [13]. Few studies focus on microbial communities in mines, and those that do are often scattered across the mining sector, geographic regions, and analytical methods. As a result, the contribution of mining microbiomes to occupational exposure and disease risk is poorly understood. Emerging conceptual frameworks, such as the environmental and occupational exposome, emphasize determinants of human health arising from long-term exposure to physical, chemical, and biological agents. Accordingly, understanding the microbial ecology of mining workplaces is necessary for effective occupational risk assessment and miner protection.

Previous studies have characterized microbial communities in individual mining environments, while reviews have addressed environmental bioaerosols, mining microbiology, or antimicrobial resistance (AMR) separately. An integrated occupational-health perspective linking the mining microbiome with microbial aerosolization, mineral-dust interactions, respiratory exposure, AMR, and environmental surveillance remains insufficiently developed. This review therefore uses the term “hidden microbiome” to describe microorganisms and microbial genetic determinants that are often overlooked in conventional mining hazard assessment. It integrates three dimensions: (i) ecological and geochemical determinants of mining-associated microbial communities; (ii) mechanisms through which mining activities generate microbial–dust exposure; and (iii) potential implications for respiratory health, AMR, and occupational risk management. Throughout the review, we distinguish environmental detection from demonstrated worker exposure, disease causation, and human transmission. The objective is to provide a critical framework for incorporating microbiological hazards into mining occupational-health assessment and surveillance.

## 2. Literature Search and Narrative Synthesis

### 2.1. Literature Search Strategy

A structured, non-systematic literature search identified published evidence on the microbiology, ecology, occupational exposure, respiratory health implications, and antimicrobial resistance (AMR) associated with mining environments. The search aimed to inform a critical narrative synthesis and bring together evidence from ecological, microbiological, occupational, clinical, and molecular studies, rather than to conduct an exhaustive systematic review or quantitative meta-analysis.

PubMed/MEDLINE, Scopus, and Web of Science were searched from database inception to August 2026. Search concepts were developed around five main themes: (i) mining environments and mining activities; (ii) microbial communities and microbiomes; (iii) bioaerosols, dust, and occupational exposure; (iv) respiratory health and opportunistic infections; and (v) AMR, resistance determinants, heavy metals, and metagenomic approaches. Representative search terms included *mining*, *mine*, *coal mine*, *metal mine*, *uranium mine*, *acid mine drainage*, *mining microbiome*, *microbial diversity*, *bioaerosol*, *dust*, *occupational exposure*, *respiratory infection*, *fungal exposure*, *antimicrobial resistance*, *antimicrobial resistance genes*, *heavy metals*, *metagenomics*, *environmental DNA*, and *occupational health*. Boolean operators were used to combine related concepts, with database-specific syntax adapted where necessary.

The search process was intentionally concept-driven and iterative. Additional relevant publications were identified by examining reference lists of key articles and reviews and by following relevant citations when these provided important mechanistic, occupational, ecological, or methodological evidence not readily captured by the initial search concepts. Priority was given to studies that provided direct evidence from mining environments or occupational populations, while relevant studies from other environmental or occupational settings were considered when they helped clarify biological mechanisms, analytical approaches, or interpretation of environmental microbial findings. The search was not intended to identify every publication addressing individual microorganisms or every occupational health outcome associated with mining. Instead, it aimed to capture the key evidence needed to examine relationships among mining-associated microbial communities, environmental conditions, microbial aerosolization, worker exposure, respiratory health, and AMR.

### 2.2. Eligibility and Selection of Literature

Publications were considered relevant when they contributed substantially to one or more of the objectives of the review. These included studies describing microbial communities in mining or mining-impacted environments; investigations of mine-associated dust, bioaerosols, water, drainage systems, sediments, or biofilms; studies addressing microbial adaptation to metals, radiation, acidity, or other mining-related environmental conditions; investigations of occupational microbial exposure and respiratory health; studies concerning opportunistic or occupational infections; and molecular or metagenomic investigations of microbial communities and resistance determinants.

We considered peer-reviewed original research, environmental microbiology studies, occupational and epidemiological investigations, outbreak reports, metagenomic studies, and relevant narrative or conceptual reviews. Publications were not considered further when they had no meaningful connection with mining environments, microbial exposure, occupational health, or the microbiological mechanisms addressed by the review. Publications were selected according to their relevance to the review objectives, the nature of the evidence provided, and their contribution to interpretation. Where evidence was limited, indirect, or primarily mechanistic, this distinction was explicitly reflected in the narrative synthesis.

### 2.3. Data Extraction and Evidence Synthesis

Information was extracted from relevant publications according to the major themes of the review. These included mining environment and geographical setting; environmental compartment; microbial groups and representative taxa; physicochemical conditions; mining activities; microbial functions and adaptations; aerosolization and potential exposure pathways; reported microbial concentrations or sequence abundances; respiratory or other health outcomes; AMR determinants; analytical methods; and important knowledge gaps.

Because this was a narrative review and did not involve primary environmental sampling, the authors did not establish mine sites, collect environmental samples, define a primary sampling schedule, or determine a primary-study sample size. Instead, characteristics of sampling conducted in the underlying studies were considered where reported. These included sampling location, environmental compartment, mine type, geographical setting, sampling duration and frequency, personal versus area-based sampling, particle size fraction, and relevant environmental conditions.

Analytical approaches in the literature included culture-based enumeration, 16S rRNA and ITS amplicon sequencing, shotgun metagenomics, environmental DNA analysis, targeted molecular assays, and particle or aerosol measurements. These methods measure different biological and analytical quantities and therefore cannot be assumed to provide directly equivalent estimates of microbial abundance or exposure. We considered differences among studies in sampling volume, sample processing, extraction procedures, molecular targets, sequencing platforms, bioinformatic pipelines, detection thresholds, and quality control procedures when interpreting their findings.

Where quantitative results were reported, they were presented in their original form when useful for illustrating the magnitude or variability of observations. We distinguished culture-based measurements such as CFU/m^3^ from relative sequence abundance and gene-level measurements obtained through molecular approaches. Quantitative findings were therefore not pooled or statistically combined. Statistical estimates, including confidence intervals, measures of dispersion, and significance tests, were reported only when available from the original studies and were not recalculated for the purposes of this review.

### 2.4. Interpretation of Evidence

The evidence was interpreted by observation type and its relevance to occupational health rather than through a formal evidence-quality or risk-of-bias scoring system. Particular attention was given to five complementary dimensions:

1. Environmental detection: Identification of microorganisms, microbial components, or genetic determinants in mining-associated environments.
2. Occupational exposure: Evidence that workers may encounter the identified biological material through inhalation, dermal contact, ingestion, or other occupational pathways.
3. Health outcome: Evidence of a defined clinical, respiratory, inflammatory, or other health outcome in exposed populations.
4. Mechanistic evidence: Experimental, molecular, or ecological evidence that supports a biological relationship.
5. Hypothesized pathway: A biologically plausible relationship for which direct occupational or epidemiological evidence remains insufficient.

These categories were used to distinguish documented observations from biological plausibility and hypothesis generation. They were not intended to constitute a validated evidence-grading system or to assign numerical quality scores. In particular, detection of microbial DNA, RNA, resistance genes, or other microbial components was not interpreted as evidence of viability, effective worker exposure, transmission, infection, or disease in the absence of supporting evidence.

### 2.5. Operational Definitions

For this review, the mining microbiome refers to microorganisms and microbial genetic material detected in mining-associated environments, including mine air, dust, rock surfaces, water, drainage systems, sediments, and biofilms. The environmental resistome refers to the collection of antimicrobial resistance genes detected within environmental microbial communities.

Microbial exposure refers to occupational contact with viable microorganisms or microbial components through inhalation, dermal contact, ingestion, or other relevant pathways. Detection of microbial DNA or resistance genes alone does not establish viability, biological activity, transmission, or disease.

The occupational exposome is considered the cumulative mixture of physical, chemical, biological, and environmental exposures encountered during mining activities. A microbial hazard refers to a microorganism, microbial component, or microbial genetic determinant with a plausible capacity to adversely affect health or contribute to environmental dissemination of AMR.

Throughout the review, three levels of observation are distinguished:

**Environmental detection:** Evidence that microorganisms, microbial components, nucleic acids, or resistance determinants are present in the mining-associated environment.

**Occupational exposure:** Evidence that workers encounter the relevant biological material at measurable or otherwise demonstrable levels.

**Health outcome:** Clinical or epidemiological evidence linking exposure with a defined health endpoint.

This distinction is central to the interpretation of the literature because environmental detection does not necessarily indicate effective human exposure, transmission, colonization, infection, or disease.

### 2.6. Limitations of the Methodological Approach

The narrative approach was appropriate because the available literature encompasses substantially different mining environments, environmental compartments, study populations, exposure measures, microbiological methods, and health outcomes. This heterogeneity limits direct quantitative synthesis for many of the questions addressed in the review. The approach allowed the review to consider ecological, microbiological, occupational, clinical, and molecular evidence together while preserving important differences between study designs and analytical methods. However, it also means that the review should be interpreted as a critical synthesis of available evidence rather than an estimate of pooled microbial prevalence, exposure, or occupational risk. Quantitative values are, therefore, presented primarily to illustrate the range, context, and variability of reported observations rather than as directly comparable estimates.

Particular caution is required when comparing culture-based measurements with amplicon sequencing, shotgun metagenomics, targeted molecular assays, or environmental DNA approaches, because these methods address different biological quantities and have different detection characteristics. The absence of standardized sampling and reporting across mining environments also limits conclusions regarding the magnitude and consistency of occupational microbial exposure.

## 3. Mining Environments as Unique Microbial Ecosystems

Mining sites are subterranean microhabitats with distinct physicochemical conditions that shape microbial colonization and interactions. These environments are characterized by low light, high humidity, variable temperature, low oxygen tension, and increased concentrations of dissolved metals and minerals, which control both community composition and ecological interactions. They provide distinct ecological niches that impose strong environmental stress on microorganisms. As a result, abundant populations of microorganisms with specialized metabolic capabilities, including metal oxidation, sulfur cycling, and biofilm formation, persist and proliferate in mines [14]. Reported microbial abundances should be interpreted cautiously because culture-based counts, amplicon relative abundances, and metagenomic read-based estimates represent different biological and analytical quantities.

Mining environments comprise metal-rich geological substrates that impose selective pressure on coexisting microbial populations. With high levels of metals such as iron, copper, uranium, and arsenic, many microorganisms have evolved strategies that allow them not only to tolerate these metals but also to use them. Microbial oxidation of sulfide minerals in mining ecosystems or acid mine drainage systems generates acidic environments [15]. These microorganisms contribute to geochemical cycling in mining ecosystems and play roles in biomining and biogeochemical metal transformations. Fungal communities also play an important role in mining microbiomes alongside bacteria. Filamentous fungi and yeasts may colonize the surfaces of rocks, dust particles from mines, and organic deposits in mining tunnels. At mining sites, genera such as Aspergillus, Penicillium, and Cladosporium are commonly monitored because they resist low concentrations of essential nutrients and environmental stress [16]. These fungi are most relevant to occupational health studies because they produce airborne spores that may aerosolize during mining and be inhaled by workers.

Mining operations, including drilling, blasting, and mechanized excavation, contribute significantly to atmospheric particulate matter loading. These particles can transport microorganisms from rock surfaces, soil remnants, or microbial biofilms in the mine environment. Viable bacterial and fungal spores that make up dust-associated microbial communities can be aerosolized into the air or disseminated over short distances along subterranean tunnels by draft wind [17]. In occupational contexts, particulate matter has also been shown to associate with microbial cells, which can protect incoming microorganisms from differences in pH and moisture availability, influence microbial persistence during transport, and increase the likelihood of respiratory deposition following inhalation.

Modern metagenomic sequencing technologies have enabled analysis of microbial communities in mining environments. Environmental DNA (eDNA) high-throughput sequencing has uncovered an unexpectedly large extent of microbial diversity in mining-impacted systems, including numerous previously uncultured bacteria and archaea [18]. These results have identified unique and complex microbial communities hosted in such mining environments, some of which are not cultivable by standard methods. Additionally, metagenomic approaches can identify metal resistance genes, stress response genes, and metabolic pathways involved in biogeochemical cycling, providing additional context for the functional potential of mining-associated organisms [19].

Quantitative evidence further illustrates the ecological heterogeneity of mining microbiomes. In coal-mine dust, Gammaproteobacteria, Alphaproteobacteria, and Betaproteobacteria have been reported to account collectively for approximately 72.5% of bacterial sequences [8], while culture-based investigations have reported airborne bacterial concentrations ranging from approximately 1.65 × 10^3^ to >20 × 10^3^ CFU/m^3^ depending on sampling location [16]. In mineralized-refuse mining environments, bacterial and fungal aerosol concentrations have reached approximately 5968 ± 1608 CFU/m^3^ and 1196 ± 370 CFU/m^3^, respectively [16]. These values should not be interpreted as directly comparable across studies because culture-based counts, amplicon relative abundances, and metagenomic measurements represent different biological quantities [8,16]. Nevertheless, the available data demonstrate substantial spatial and environmental variation in microbial exposure [16]. Geochemical conditions, including pH, metal concentration, redox state, moisture, organic-carbon availability, and oxidative stress, provide important selective pressures that contribute to this variation.

From an occupational health perspective, these ecological adaptations matter because the same environmental conditions that select specialized microbial communities can influence their persistence in dust, water, and biofilms, and their subsequent aerosolization during mining activities. However, ecological abundance or adaptation does not necessarily translate into increased worker health risk. Occupational relevance depends on whether microorganisms or their biological components become airborne, remain viable or biologically active, reach workers at meaningful exposure levels, and are associated with adverse health outcomes.

## 4. Microbial Diversity in Coal, Uranium, and Metal Mines

Coal mines are among the best-known environments for studying microbial diversity. These systems contain abundant organic carbon substrates, methane gas, and particles from excavated material [20]. Proteobacteria, Firmicutes, Actinobacteria, and Bacteroidetes are frequently reported as major bacterial groups in coal-mine environments. Bacteria from these phyla play major roles in carbon cycling, degrading organic matter and converting it into humus, or soil organic matter, which serves as a habitat for microorganisms such as bacteria and fungi [21].

Methanogenic archaea are also commonly found in coal seams and underground coal mines, where they use anaerobic metabolic pathways to produce methane. Working and living as part of complex microbial communities, these organisms collectively decompose coal-derived organic compounds and generate molecular hydrogen, which other fermentative microorganisms consume via methanogenesis. Methanogenic archaea, including *Methanobacterium* and *Methanosarcina*, have been detected in coal seams and underground coal environments and may contribute substantially to anaerobic carbon cycling. However, their relative abundance varies among sites and analytical approaches, and the available evidence does not support describing these taxa as consistently dominant across coal-mining environments [22]. Another location from which fungal communities can be sampled is within the mine itself, where dust particles can serve as niches for these fungi. Fungal spores can persist within dust particles. Many types of dust, including mine dust, can harbor high populations of fungi. Fungal genera known to be present in the air include Aspergillus, Penicillium, and Cladosporium, which are also usually found in underground coal mines and can be aerosolized during coal cutting [23].

Uranium mines are a unique microbial habitat, with high concentrations of radioactive isotopes and heavy metals. Microorganisms here are also often highly resistant to radiation and to metal poisoning, allowing some taxa to persist under conditions characterized by elevated radiation, heavy metal exposure, oxidative stress, low pH, or nutrient limitation [24]. Microbial communities adapted to extreme conditions (oxidative stress, heavy metal toxicity, low pH, and nutrient scarcity) occur in uranium and metal mining environments. Uranium becomes more or less soluble depending on its oxidation state, which can be reduced to more insoluble forms through biogeochemical processes driven by metal-resistant bacteria prevalent in uranium mining areas that are abundant and participate massively in biogeochemical cycling, such as reduction and immobilization of uranium species, and that may have potential applications in the bioremediation of contaminated sites [25]. Heavy-metal–resistant fungi can also influence mineral weathering and metal transformation via biosorption and biomineralization, changing geochemical dynamics in uranium-contaminated environments [26]. Metallophilic and acidophilic microorganisms are adapted to extreme habitats, including metal-rich mining sites such as copper, gold, and iron ore mines. Iron- and sulfur-oxidizing bacteria, such as *Acidithiobacillus ferrooxidans*, *Leptospirillum ferrooxidans* species, and *Sulfobacillus* species, oxidize sulfide minerals and produce acid mine drainage. In addition, these microorganisms are exploited industrially in bioleaching processes that can recover metals from low-grade ores and mining tailings [27].

In contrast, mines with ore at neutral or alkaline pH can harbor distinct microbial consortia with different metabolic properties [28]. Mining operations and human activities can also affect microbial community composition. Changes in ventilation systems, water treatment methods, and the artificial breakdown of geological strata alter mine conditions, affecting the initiation and development of microbial colonization. These factors may influence the spatial distribution and development of microbial communities within metal mine workings [29]. Mining environments support distinct microbial communities shaped by geological, geochemical, and operational conditions. Coal, uranium, metal mining, acid mine drainage, and mine dust environments harbor different microbial groups with specialized adaptations and ecological functions, while mining activities can also facilitate microbial aerosolization and occupational exposure. Table 1 summarizes major microbial groups, representative taxa, environmental drivers, ecological functions, and potential occupational relevance across major mining environments.

Across mining types, the dominant microbial communities reflect differences in geochemistry and operational conditions, while the principal occupational exposure pathways converge on inhalation of dust-associated microorganisms, aerosols, and microbial components. Thus, mining type influences both microbiome composition and the characteristics of potential biological exposure, but environmental detection alone does not establish an equivalent occupational health risk across these settings.

## 5. Bioaerosols and Dust-Associated Microbiomes in Mining Environments

Mining environments have several environmental reservoirs for bioaerosol generation. Microbial biofilms consisting of bacteria and fungi can survive under extreme geochemical conditions on rock surfaces and mine walls. Microorganisms buried in the ground can be released into the air as airborne particulates when mining operations disturb them [30]. In addition, mine water systems and drainage canals are well known to host diverse microbiomes that can become aerosolized through splashing, pumping, or ventilation airflow [31]. Dust generated by mining operations is a key vector for inhalation exposure to airborne microbes, releasing them from the natural environment. Drilling and blasting operations generate particulates that can carry soil debris, mineral substrates, and microbial biofilm, including microorganisms. Airborne dust can transport bacterial and fungal spores and facilitate their dispersal through underground mine environments, helping them spread into the subsurface workplace. Respirable dust particles can penetrate deep into the human respiratory tract, allowing dust-associated microbial cells to contribute to occupational respiratory exposure [32].

Environmental conditions, mining activities, and geological formations also influence the microbial composition of mine bioaerosols. Microbial exposure in mining settings has shown a varied composition of airborne microorganisms, including bacterial families from the phyla Proteobacteria, Firmicutes, and Actinobacteria, as well as fungal genera such as Aspergillus, Penicillium, and Cladosporium [17]. Anaerobic microbes are commonly found in occupational air samples and originate from both the environment and mining activities. In addition to living microorganisms, mine bioaerosols contain many microbial components, including endotoxins and various fungal cell wall fragments. Gram-negative bacterial endotoxins induce inflammatory responses in the airway and have been shown to contribute to respiratory symptoms in workers exposed to organic dust environments [33]. Likewise, inhaling fungal spores and fragments can act as allergens or immune triggers, inducing hypersensitivity responses after repeated airborne exposure.

Molecular techniques, particularly metagenomic and amplicon sequencing, have recently revealed greater diversity in airborne microbial communities than previous culture-based approaches, enabling cross-environment comparisons [34]. These methods have enabled the detection of numerous hard-to-cultivate microbes, providing a more complete picture of microbial diversity in occupational environments.

Dust-associated microbiomes represent a plausible occupational pathway through which mining workers may encounter airborne microorganisms and microbial components. Although environmental and area-based measurements demonstrate microbial material in mining dust and bioaerosols, direct personal-exposure measurements linking these microbial exposures to individual workers remain limited. Accordingly, the relative contribution of dust-associated microorganisms to total occupational microbial exposure cannot currently be quantified with confidence. Microbial cells and spores form complex associations with respirable dust particles released during mining activities, which can carry airborne, widely dispersed particle–microbe clusters for long periods [17]. These clusters may enable microbial dispersal through subsurface mine networks via airflow currents and ventilation systems. Furthermore, mineral dust–microbial cell interactions are hypothesized to support microbes’ survival and persistence during atmospheric transit. Therefore, mineral particles protect microbes by forming a physical barrier against environmental stressors, including ultraviolet radiation, desiccation, and oxidative damage. As a result, attached microbes may persist longer than free-living microbial cells under some environmental conditions [35]. This protective association may enhance microbial persistence during aerosol transport and could increase the likelihood of respiratory deposition following inhalation; however, evidence demonstrating subsequent colonization or infection among exposed miners remains limited.

Engineering approaches that reduce respirable dust may also reduce the aerosolization and transport of dust-associated microbial material. Recent advances include multi-scale droplet atomization for respirable dust capture, surfactant-based enhancement of coal-dust wettability and droplet coagulation, and the molecular design of surfactants to improve dust-control efficiency [36,37,38]. However, these technologies have primarily been evaluated for particulate-dust reduction, and their effectiveness in reducing viable microorganisms, microbial components, or microbial genetic material remains to be established.

Occupational health studies show that chronic exposure to microbial components in dust can lead to respiratory symptoms and inflammation in workers exposed to high levels of airborne dust. In mining environments, where workers inhale high levels of respirable particulates during daily work, microbial components in dust particles represent an additional biological hazard that may also interact with traditional exposures to mineral dust [39].

**Figure 1:**
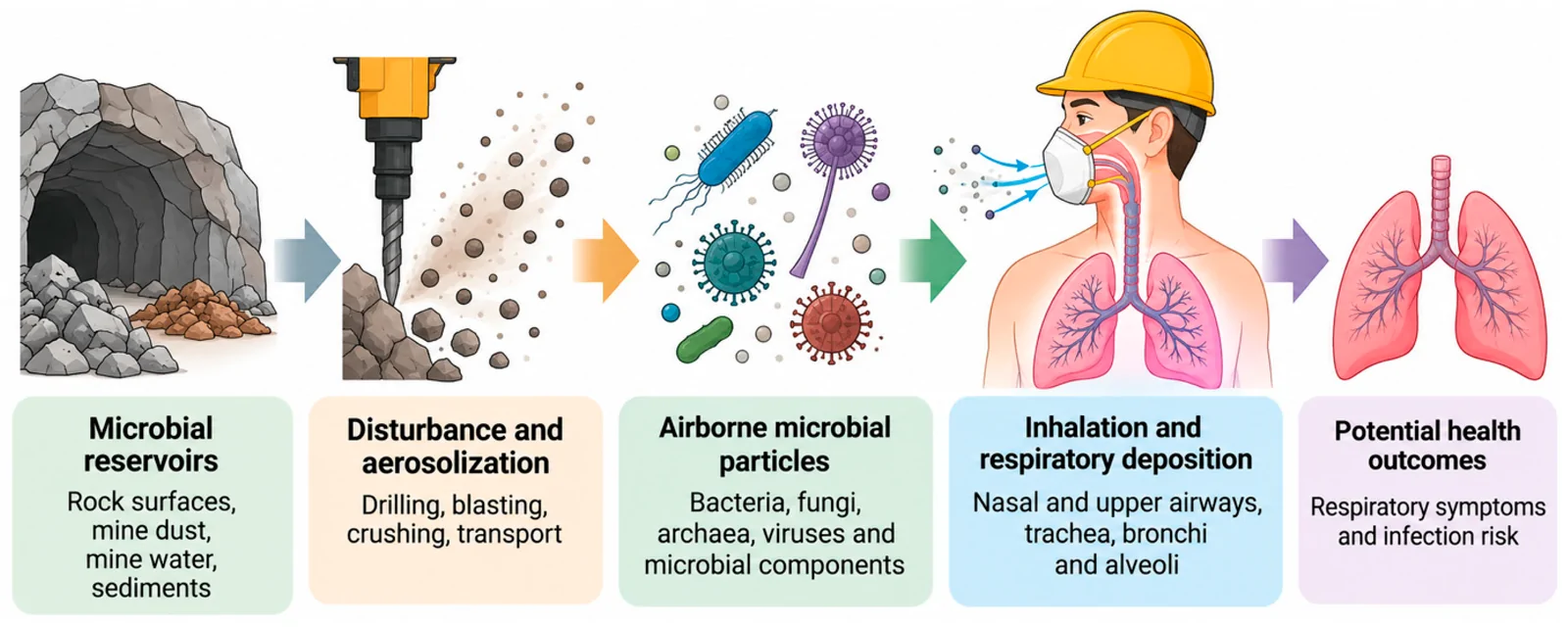
Conceptual pathways of microbial aerosol exposure in underground mining environments. Mining activities generate dust that may carry microorganisms originating from rock surfaces, mine water, and biofilms. Dust-associated microbial material can become aerosolized and transported through ventilation systems, creating plausible inhalation exposure pathways. The pathways shown represent potential exposure routes and do not establish disease causation. The authors conceptually designed the figure using BioRender (available online at https://www.biorender.com; accessed on 22 May 2026) and Adobe Illustrator version 28.5 based on mechanisms described in the published literature.

Bioaerosol concentration and composition are influenced by various environmental and operational conditions that characterize mining environments. Ventilation systems in underground tunnel environments are essential for controlling airborne particulate matter and bioaerosols, as inadequate ventilation raises concentrations of respirable dust and microorganisms, increasing occupational exposure and respiratory health risks among workers [40]. Effective ventilation, by contrast, can reduce airborne microbial loads by diluting and removing contaminated air [41]. High humidity in mines can support microbial growth on surfaces, prolong the survival of airborne microbes, and increase the introduction of surface particles into the air; however, airflow patterns in mine tunnels dictate how aerosolized particles are distributed [42]. Fluctuations in airborne microbial concentrations can also occur due to seasonal variations and changes in mining operations. Figure 1 provides a conceptual overview of potential sources and exposure pathways for mining-related bioaerosols.

Available evidence shows considerable variation in the composition and distribution of mining-associated microorganisms by mine type, environmental conditions, mining activities, ventilation, and sampling approach [43]. Although culture-based and molecular studies have identified diverse bacterial and fungal communities in mine dust and bioaerosols, differences in sampling and analytical methods currently limit direct quantitative comparison of microbial exposure across mining environments. Quantitative investigations demonstrate substantial spatial variation in mining-associated bioaerosols. In an underground hard-coal mine, culture-based measurements showed mesophilic bacterial concentrations ranging from approximately 1.65 × 10^3^ CFU/m^3^ to more than 20 × 10^3^ CFU/m^3^, depending on sampling location. The same investigation identified substantial variation in bacterial and fungal community composition between underground locations [29]. In another mining-related environment involving mineralized refuse, bacterial and fungal aerosol concentrations reached approximately 5968 ± 1608 CFU/m^3^ and 1196 ± 370 CFU/m^3^, respectively [16]. However, these measurements should not be directly compared with sequencing-derived relative abundances because culture-based counts and molecular abundance estimates represent different biological and analytical quantities. Because quantitative microbial measurements are reported in heterogeneous units and using different analytical approaches, Table 2 presents the values as reported rather than pooling or ranking them across mining environments.

Although microbial concentrations have been reported in mining aerosols, direct comparison with other occupational environments remains difficult because studies differ in sampling volume, particle size fraction, culture media, molecular targets and reporting units. Accordingly, CFU/m^3^ values from mining environments should not be interpreted as directly equivalent to sequencing-derived relative abundance or gene-copy measurements from other occupational settings. Where standardized culture-based measurements are available, cross-occupational comparisons may nevertheless provide useful context for determining whether mining represents a distinctive microbial exposure environment.

### 5.1. Microbe–Mineral Dust Interactions and Potential Synergistic Toxicity

Mineral dust and microbial constituents should not necessarily be considered independent occupational exposures. Microorganisms, spores, and microbial products can associate with respirable particles, potentially influencing persistence, transport, and respiratory deposition. Following inhalation, mineral particles and microbial components may activate overlapping inflammatory pathways. Endotoxin from Gram-negative bacteria can stimulate innate immune responses, whereas fungal β-glucans can activate Dectin-1-associated signaling. These mechanisms provide a biologically plausible basis for interaction between particulate and microbial exposures. However, this mechanistic interpretation is not equivalent to evidence of additive or synergistic toxicity in mining workers. The available mining literature does not establish a quantitative interaction effect between microbial components and mineral dust, and direct dose–response studies combining these exposures remain scarce. Accordingly, additive or synergistic toxicity should be treated as a testable hypothesis rather than an established feature of mining exposure.

### 5.2. Implications for Engineering Control of Microbial–Dust Exposure

Because microbial material can associate with respirable dust, interventions that reduce respirable particulate matter could potentially reduce the physical transport of associated microbial material; however, this effect has not been directly demonstrated in mining microbiome studies. Current engineering approaches in underground mining include water-based suppression, optimized droplet atomization, ventilation management, and surfactant-assisted dust wetting. Recent studies have investigated multi-scale droplet atomization for respirable dust suppression, surfactant-mediated enhancement of coal-dust wettability, and surfactant-tail engineering to improve dust control in underground environments. These approaches primarily target particulate control rather than microbial hazard reduction; therefore, their effectiveness in reducing viable microorganisms, microbial DNA, or microbial inflammatory components should be evaluated directly.

At present, no generally accepted dose–response relationship has been established between mining-associated microbial burden and respiratory outcomes. This limitation reflects differences in how researchers measure viable microorganisms, microbial DNA, endotoxin, fungal β-glucan, and respirable particulate matter. Future studies should quantify these exposures concurrently with standardized respiratory outcomes and mineral dust measurements to determine whether microbial and particulate exposures exert independent, additive, or synergistic effects.

## 6. Respiratory Health Effects and Opportunistic Infections Associated with Mining Microbiomes

### 6.1. Noninfectious Respiratory Effects

Hypersensitivity pneumonitis (HP), also known as extrinsic allergic alveolitis, is an immune-mediated inflammatory lung disease caused by recurrent inhalation of microbial antigens. These antigens, produced by bacteria, fungi (including fungal spores), or actinomycetes, are associated with occupational exposure. Hypersensitivity reactions to organic materials have also been linked to microbial-particulate complexes ([52]). Although microbial particles in mine dust can be inhaled and deposited in the respiratory tract, evidence directly linking long-distance airborne transport from mining sites to specific respiratory infections remains limited.

Thermophilic actinomycetes, particularly species in the genera Streptomyces and Saccharopolyspora, are known agents of hypersensitivity pneumonitis associated with occupational exposure to organic dust. Although these organisms are more commonly associated with agricultural environments, they can also be found in mine dust and in organic deposits within underground tunnels. These conditions involve immune responses in the alveoli, triggered by exposure to microbial spores or cell fragments, leading to cough, shortness of breath, and reduced lung function [53]. Bioaerosols in mines can also contain fungal spores associated with hypersensitivity and allergic respiratory diseases.

### 6.2. Opportunistic and Occupational Infections

*Aspergillus* spp. are commonly detected in airborne and dust-associated environments and can cause allergic or invasive disease in susceptible individuals; however, the presence of *Aspergillus* DNA or spores in mining environments does not by itself establish mining-acquired aspergillosis. In contrast, evidence directly demonstrating occupationally acquired aspergillosis attributable to mining exposure remains limited. Fungal genera, namely Aspergillus and Penicillium, are ubiquitously present in mining environments and may release spores that induce allergic bronchopulmonary reactions, respiratory irritation (including asthma-like symptoms), and allergic inflammation in exposed workers [54]. Beyond allergy-driven pathology, mining aerosols can harbor opportunistic fungal pathogens that can infect susceptible hosts. Among opportunistic pathogens, Aspergillus species are particularly significant and among the most prevalent airborne opportunistic fungi, with inhaled conidia causing disease ranging from allergic bronchopulmonary aspergillosis to invasive pulmonary aspergillosis [55]. Mining-related environmental disturbances can aerosolize fungal spores from disturbed soil or sediment, increasing inhalation of pathogens such as Histoplasma capsulatum, which is associated with histoplasmosis [56]. While histoplasmosis is most often associated with bat or bird droppings, excavating contaminated soils can likewise aerosolize infectious spores [56]. Other yeasts, such as species of Cryptococcus, have also been identified in dusty, polluted environments, and pulmonary cryptococcosis can result after inhalation of these fungi by individuals with immune deficiency; however, evidence linking mining exposure to cryptococcal infection remains limited [57]. Nontuberculous mycobacteria (NTM) are obligate soil- and water-borne environmental mycobacteria that have increasingly been reported as opportunistic human pulmonary pathogens. During work activities, workers may inhale these organisms, especially when water aerosols or contaminated dust are used [58]. Microbial compounds (e.g., endotoxins from Gram-negative bacteria) may mediate inflammatory responses in the respiratory tract. Occupational exposure to organic dust is associated with airway irritation, fever, and decreased lung function due to endotoxins. Endotoxin exposure has been associated with exacerbation of pre-existing respiratory disease or chronic lung inflammation in mining environments, where microbial particulates are bound to respirable dust [47]. Moreover, microbial cells and spores attached to dust particles may serve as carriers that deposit deeply in the respiratory tract. Mining workers may experience co-exposure to mineral dust and microbial agents, although the independent contribution of microbial exposure to respiratory disease remains uncertain [48]. Understanding this relationship is critical to establishing multi-partite occupational health strategies that address both chemical and biological hazards in mining environments. Mining-associated bioaerosols may contain bacteria and fungi with potential respiratory or opportunistic pathogenic relevance. However, evidence varies considerably among microorganisms, ranging from documented occupational outbreaks to environmental detection without demonstrated disease causation. For example, occupational histoplasmosis has been associated with inhalation of aerosolized *H. capsulatum* spores following disruption of contaminated soil or bat/bird guano, including in enclosed tunnel and mining-related environments, whereas evidence for *Aspergillus*, *Pseudomonas*, and nontuberculous *Mycobacterium* spp. in mining and related extractive settings is more commonly based on environmental detection and occupational exposure studies rather than well-established occupational disease associations [49,59]. Table 3 summarizes the microorganisms, exposure pathways, associated health outcomes, and strength of available evidence.

Evidence linking mining-associated microorganisms to occupational disease currently falls into three broad categories. First, some specific environmental exposures, particularly Histoplasma-contaminated soil or bat guano in tunnels, have been associated with documented outbreaks among workers. Second, occupational studies have demonstrated exposure to microbial components such as endotoxin and fungal material, but these studies do not necessarily establish that the microorganisms themselves caused disease. Third, metagenomic studies frequently identify opportunistic pathogens or virulence-associated taxa, but environmental detection alone cannot establish infectivity, viability, host exposure dose, or clinical causation. Accordingly, the presence of Aspergillus, Pseudomonas or Acinetobacter in mining environments should be interpreted as evidence of potential biological exposure rather than proof of occupational infection.

Overall, the epidemiological evidence is strongest for specific exposure scenarios such as Histoplasma-contaminated soil or bat-associated material in underground environments, whereas evidence for most other mining-associated microorganisms remains based predominantly on environmental detection, exposure characterization or biological plausibility. This distinction limits causal inference about the mining microbiome’s contribution to respiratory disease.

A major limitation of the current evidence base is that most studies identify microorganisms or microbial components in mining environments without simultaneously measuring individual worker exposure and subsequent health outcomes. Consequently, detection of potentially pathogenic taxa should be interpreted primarily as evidence of a potential hazard rather than evidence of attributable occupational disease. Establishing causal relationships will require prospective studies incorporating personal exposure measurements, microbial viability or activity, relevant biomarkers, respiratory outcomes, and appropriate adjustment for established mining hazards such as silica, metals, and tobacco exposure.

## 7. Emerging Pathogens and Antimicrobial Resistance in Mining Microbiomes

Microbial communities living under varying conditions, such as high metal concentrations, oxidative stress, or limited nutrient availability, are prevalent in subsurface environments (such as mines). Such selective pressures may favor microorganisms with adaptive mechanisms that enhance survival under environmental stress. Many of these mechanisms, including efflux pumps, metal resistance genes, and stress response systems, have been linked to microbial pathogenicity and AMR [29]. Microorganisms in mining environments include genera such as Pseudomonas, Acinetobacter, and Burkholderia, which are well-known opportunistic pathogens in clinical settings [30]. These organisms can occupy diverse environmental niches and are biologically capable of causing infection in susceptible hosts, but the available mining-specific evidence does not establish that environmental detection in mining aerosols results in occupational infection [46]. Thus, occupational exposure to environmental microorganisms in mining ecosystems should currently be regarded as a potential biological exposure pathway rather than an established pathway for pathogen transmission. Importantly, microbial biofilms in mining environments may provide conditions conducive to genetic exchange. Horizontal gene transfer in biofilm communities occurs through conjugation, transformation, and transduction [61]. These processes can transfer resistance genes and virulence-associated determinants between microbial strains, potentially contributing to their dissemination within environmental microbial communities.

Chronic exposure to heavy metals in mining environments can exert strong selective pressure on microorganisms inhabiting metal mine tailings, drainage waters, and mineral substrates, favoring the persistence of metal-resistant populations [29]. Studies have reported co-occurrence between metal resistance genes (MRGs) and antibiotic-resistance genes (ARGs) in mining-impacted environments [62]. However, the strength of evidence for physical genetic linkage varies among studies. Short-read metagenomic co-occurrence generally indicates statistical association rather than physical linkage, whereas long-read sequencing, complete genome reconstruction, or experimentally validated mobile genetic elements can provide stronger evidence that ARGs and MRGs occur within the same genome or genetic element. Accordingly, the terms ‘co-occurrence’, ‘genetic linkage’, and ‘demonstrated horizontal transfer’ should not be used interchangeably. Where metal- and antibiotic-resistance determinants are genetically linked or co-regulated, metal exposure may contribute to the co-selection and persistence of pre-existing antibiotic-resistance determinants, including in environments with limited antibiotic exposure.

The environmental resistome comprises the collection of antimicrobial resistance genes present within environmental microbial communities. Improvements in metagenomic sequencing have enabled researchers to study the diversity and distribution of resistance genes in ecosystems, including mining sites [63]. These studies found that mining-associated microbes often harbor diverse resistance determinants, including β-lactams, tetracyclines, and aminoglycosides, among other antibiotic classes. Metagenomic analyses of mine drainage and contaminated sediments have revealed genes associated with environmental bacteria and potential opportunistic pathogens [64]. These findings support environmentally relevant resistance reservoirs in mining-impacted ecosystems.

**Figure 2:**
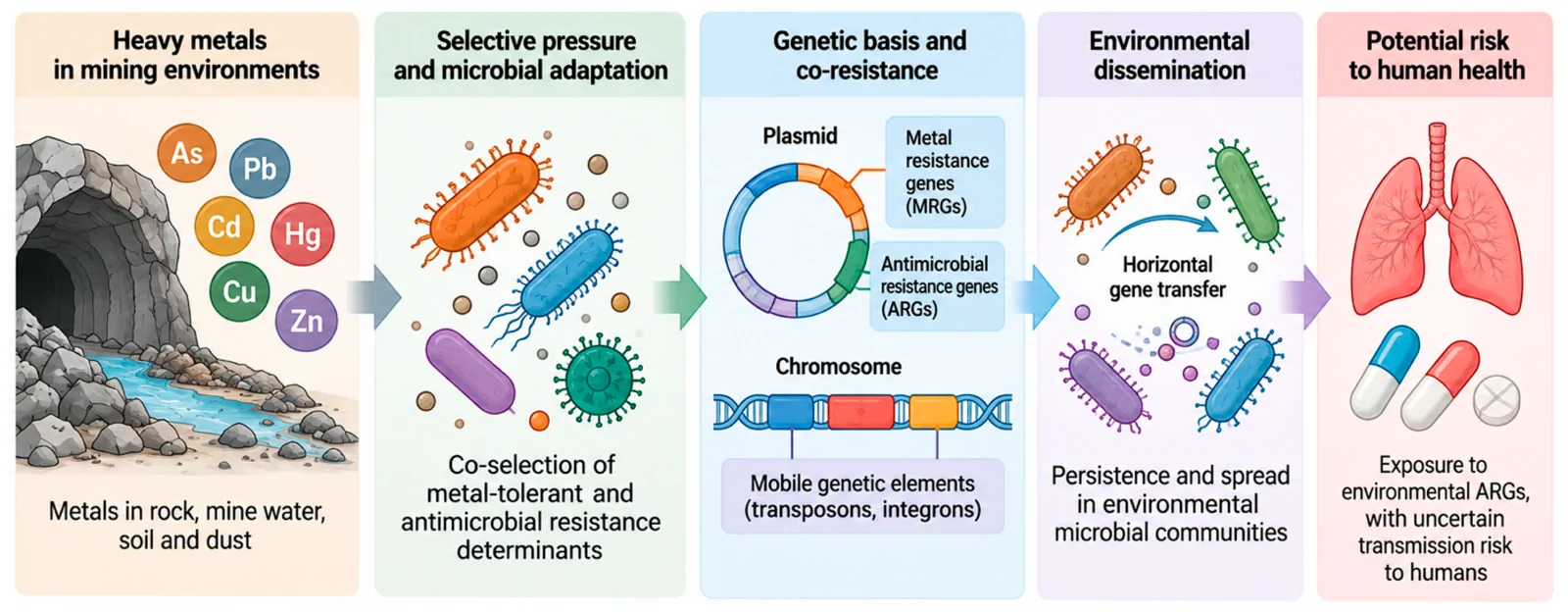
Proposed mechanisms of metal-associated co-selection of antimicrobial resistance determinants in mining environments. Heavy metal exposure can select for metal-resistant microorganisms, while genetic linkage or co-regulation of metal- and antibiotic-resistance determinants may contribute to co-selection. Mobile genetic elements may facilitate horizontal gene transfer within environmental communities. The framework represents a plausible environmental mechanism and does not establish transmission to workers or clinical pathogens. The authors conceptually designed the figure using BioRender (available online at https://www.biorender.com; accessed on 22 May 2026) and Adobe Illustrator version 28.5, based on mechanisms described in the published literature.

Overall, mining environments may represent reservoirs of ARGs and metal resistance determinants, and co-selection and horizontal gene transfer provide plausible mechanisms for their environmental persistence and dissemination. However, detection of ARGs or their association with mobile genetic elements does not establish active transmission to miners, human-associated microbiomes, or clinical pathogens. Establishing their occupational significance will require longitudinal studies integrating environmental resistome profiling with personal exposure measurements, worker microbiological or microbiome sampling, and genomic comparison of environmental and human isolates. Future studies should combine environmental resistome profiling with personal exposure measurements, longitudinal worker microbiome sampling, and genomic comparison of environmental and human isolates. Figure 2 summarizes mechanisms of metal exposure, adaptive evolution, and resistance in these environments.

## 8. Metagenomics and Environmental Surveillance of Mining Microbiomes

Culture-based assays for isolating and identifying microorganisms have been a cornerstone of traditional microbiological investigations in occupational and environmental settings. While these approaches remain useful for identifying living organisms and studying microbial physiological properties, they typically recover only a fraction of microbial diversity in environmental samples. Environmental microorganisms often exist as viable but non-culturable or have growth requirements that laboratory media cannot provide [65]. Consequently, culture-dependent assessments of airborne microorganisms in mineralized environments may underestimate both microbial abundance and taxonomic richness. The consequences for occupational health research could be significant; conventional detection methods may miss pathogenic or allergenic microorganisms.

More recently, metagenomics has emerged as a powerful approach for characterizing complex microbial communities in environmental ecosystems. In these applications, eDNA can be extracted and sequenced directly from environmental samples such as mine dust, bioaerosols, sediments, and draining waters. This approach bypasses the need for prior cultivation for organism characterization and works for both already characterized organisms and new species detected solely in environmental samples [66]. Amplicon-based sequencing is widely used in microbiome studies, and two major sequencing strategies are commonly applied. Amplicon sequencing focuses on conserved genes to identify microbial taxa, such as the 16S rRNA gene for bacteria or the internal transcribed spacer (ITS) region for fungi, in environmental samples [67]. It is a relatively low-cost way to characterize microbial community composition and relative abundance.

In contrast, shotgun metagenomic sequencing characterizes the genetic content of environmental DNA and can identify sequences associated with metabolic pathways, virulence-associated determinants, and antimicrobial resistance. These findings provide information about the functional potential encoded within the sampled microbial community but do not, by themselves, demonstrate gene expression, protein activity, phenotypic antimicrobial resistance, or expressed virulence [67]. Functional interpretation, therefore, requires complementary approaches, such as metatranscriptomics, proteomics, culture-based phenotypic testing, or other targeted functional assays, depending on the research question. The interpretation of sequencing results depends on the analytical approach. Amplicon sequencing primarily provides taxonomic profiles and relative abundance estimates for targeted microbial groups, whereas shotgun metagenomics provides broader information on environmental DNA and encoded functional potential. Neither approach alone establishes microbial viability or phenotype. Culture-based methods can provide evidence of viable organisms and permit phenotypic testing but may underestimate organisms that are difficult to cultivate. Integrating these approaches therefore provides a more informative assessment than relying on any single method.

Despite its advantages, metagenomic sequencing has important limitations for assessing occupational microbial risk. Detection of microbial DNA does not establish viability, infectivity, or active gene expression. Short-read sequencing may also make it difficult to determine whether ARGs and metal resistance genes occur within the same organism or mobile genetic element. Sequencing depth, extraction efficiency, and database composition can influence relative abundance estimates, while differences in bioinformatic pipelines may produce substantially different taxonomic and functional profiles. Long-read and genome-resolved approaches may improve linkage between resistance genes and their microbial hosts, but these methods remain more resource-intensive. Consequently, metagenomic findings should be integrated with culture, quantitative molecular assays, and epidemiological data rather than interpreted as direct evidence of occupational disease or transmission.

Monitoring microbial communities in environmental contexts has increasingly relied on eDNA analysis. In mining environments, researchers can recover eDNA from air filters, dust particles, water systems, and surface biofilms to detect microbial taxa [68]. This technique enables researchers to identify microbial organisms that may not be easily cultured in the laboratory. Molecular approaches can improve understanding of the composition and temporal dynamics of mine-associated bioaerosols through airborne microbial monitoring. For example, higher airborne microbial abundance in agricultural settings is associated with microbes from agriculture-related soil amendments (e.g., fertilizers) and with the aerial dispersal of manure from sprinkler irrigation systems, which promote the wind transport of soil particles [6]. Such data can be used to identify times or places with a higher risk of microbial exposure.

Recent technological developments have improved environmental microbial surveillance analytics. Portable sequencing technologies, including nanopore sequencing platforms, now enable rapid sequencing of microbial DNA directly in the field. These portable systems permit real-time testing of environmental samples, which could facilitate the rapid identification and quantification of microbial threats in occupational settings [69].

### Proposed Framework for Mining Microbiome Surveillance

A practical mining microbiome surveillance framework should integrate environmental sampling, exposure assessment, microbiological characterization, health monitoring, and risk management. First, collect samples from mine dust, ventilation air, rock surfaces, mine water, drainage systems, and biofilms across different locations, mining activities, and time periods. Second, use personal and area-based sampling to characterize worker exposure, alongside measurements of respirable dust and relevant physicochemical parameters. Third, complement culture-based methods with 16S rRNA/ITS sequencing, shotgun metagenomics, and targeted molecular assays to characterize microbial communities and detect potentially pathogenic taxa and AMR determinants. Fourth, integrate microbiological findings with occupational health data, including respiratory symptoms and pulmonary function, to distinguish environmental detection from clinically relevant exposure. Standardized sampling, sequencing, and bioinformatic protocols will be essential for generating comparable exposure benchmarks across mining environments.

Implementation should be phased according to available resources and risk. Routine surveillance could initially use culture-based methods and targeted quantitative molecular assays, with shotgun or portable sequencing reserved for research, outbreak investigation, or sites with unusual microbial or resistance profiles. Integration with existing occupational monitoring should combine microbial measurements with respirable dust, ventilation, metal exposure, respiratory symptoms, and pulmonary function data. Standardized sampling frequency, quality control procedures, metadata collection, sequencing pipelines, and reporting criteria will be necessary to ensure comparability, while cost, laboratory capacity, personnel expertise, and data-processing requirements should guide surveillance method selection.

## 9. Knowledge Gaps, Research Priorities, and Future Directions

Despite increased awareness of mining-associated microbial complexity, major knowledge gaps remain. Research is spatially fragmented and often focused on individual mine sites or specific extraction systems. Differences in geology, mining methods, ventilation, climate, sampling strategy, and analytical methods limit the generalizability and direct comparison across studies. Comparative, multicenter investigations are therefore needed to determine whether reproducible microbial signatures and exposure patterns occur across mining environments. To avoid overestimating potential health risks, we organized the available evidence according to the type of support for occupational exposure, biological effects, and disease causation (Table 4). This distinction matters because microorganisms and AMR genes have been detected in mining environments, but direct epidemiological evidence linking these findings to disease or transmission among miners remains limited.

This framework highlights an important distinction between hazard identification and demonstrated health risk. For example, detecting potential pathogens or resistance genes in mine dust, water, or bioaerosols indicates an environmental reservoir but does not establish microbial viability, effective human exposure, transmission, or disease causation. Current evidence for direct transmission of resistant microorganisms from mining environments to humans remains scarce, supporting the need for longitudinal environmental and occupational epidemiological studies [70,71]. Across the available literature, microbial findings vary substantially according to mining environment, environmental compartment, sampling strategy, analytical method, and operational conditions. Culture-based investigations may provide quantitative measurements such as CFU/m^3^, whereas amplicon sequencing and shotgun metagenomics generally report relative sequence abundance or gene-level measurements. These measurements represent different biological quantities and should therefore not be directly pooled or ranked. Table 5 summarizes representative findings across mining-related environments to illustrate this methodological and ecological heterogeneity.

The comparison highlights substantial methodological and ecological heterogeneity among mining studies. Reported microbial concentrations and community profiles are influenced by where, when, and how samples are collected and analyzed. Consequently, differences between studies should not automatically be interpreted as differences in occupational risk. Standardized sampling and analytical approaches, together with personal exposure measurements and longitudinal health assessments, are needed to determine whether environmental microbial patterns translate into meaningful occupational exposure or health effects.

A major gap is the limited availability of longitudinal datasets linking microbial exposure measurements with worker health outcomes. Most studies identify microorganisms or microbial components without simultaneously measuring individual exposure, viability, or biological activity, and subsequent clinical outcomes. Future occupational studies should therefore integrate personal and area-based microbial measurements with respirable dust, metals, chemical exposures, biomarkers, respiratory symptoms, and pulmonary function while accounting for established confounders. Prospective multicenter cohorts would be particularly valuable for determining whether microbial exposures contribute independently or synergistically to respiratory disease.

Advances in metagenomics, environmental DNA analysis, and portable sequencing provide increasingly powerful tools for characterizing mine dust, ventilation air, drainage water, and associated resistomes. Their occupational value will depend on standardized sampling, quantitative exposure assessment, quality control, genome-resolved analysis, and functional validation of resistance determinants. Priority areas include longitudinal monitoring of bioaerosols, establishing baseline microbial and resistome profiles across mining environments, assessing microbial viability and activity, and conducting prospective studies linking environmental and personal exposure measurements with health outcomes. An occupational-exposome framework can then integrate microbial exposures with mineral dust, metals, toxic gases, heat, humidity, and other workplace stressors.

Another unresolved question is whether microbial components and mineral dust exert additive, synergistic, or antagonistic effects on respiratory health. Existing studies have generally evaluated particulate matter, silica, or microbial bioaerosols separately. Future work should use longitudinal cohorts and, where appropriate, factorial experimental designs to distinguish the effects of respirable mineral dust, microbial biomass, endotoxin, fungal β-glucan, and metal exposure. Future investigations should move beyond cross-sectional environmental characterization toward longitudinal, multicentre occupational studies. Such studies should combine area-based and personal microbial exposure measurements with respirable dust, metals, ventilation parameters, microbial viability or activity, biomarkers, respiratory symptoms, pulmonary function, and, where appropriate, microbiological or clinical outcomes. Larger study populations and repeated measurements across seasons, mining activities, and environmental conditions will be important for determining exposure variability, establishing dose–response relationships, and distinguishing environmental presence from clinically relevant occupational exposure.

## 10. Conclusions

Mining environments host diverse microbial communities shaped by geochemical conditions and mining activities, and mining operations can aerosolize microbial cells, spores, and microbial components together with mineral dust. Current evidence supports the presence of a previously underrecognized mining microbiome and identifies plausible pathways for biological exposure and environmental AMR persistence. However, direct epidemiological evidence linking mining-associated microbial exposure to disease and direct transmission of mining-derived resistance determinants to workers remains limited. Environmental and metagenomic detection should therefore be interpreted as evidence of presence or functional potential, not proof of viability, infectivity, transmission, or disease causation.

## 11. Limitations

This review has several limitations. First, the available evidence is heterogeneous with respect to mining type, geographic setting, environmental compartment, sampling strategy, sampling duration, particle size fraction, and analytical method. Second, many studies rely on area-based environmental measurements rather than personal worker exposure measurements, limiting assessment of individual exposure. Third, environmental conditions such as ventilation, humidity, temperature, geochemistry, mining activity, and seasonal variation can influence microbial measurements and reduce comparability between studies. Fourth, culture-based, amplicon sequencing, metagenomic, and targeted molecular methods measure different biological quantities and have different detection limitations. Fifth, many studies do not simultaneously assess microbial viability or activity, quantitative exposure, and worker health outcomes. Finally, the limited availability of longitudinal and multicentre occupational studies restricts causal inference and generalizability.

## Figures and Tables

**Table 1:** Comparative microbial, ecological, and occupational characteristics of major mining environments.

Mining Environment	Major Microbial Groups	Representative Taxa	Major Environmental Drivers	Ecological Functions/Adaptations	Major Occupational Exposure Pathways	Occupational Interpretation	References
Coal mines	Bacteria, methanogenic archaea, fungi	*Pseudomonas*, *Bacillus*, *Methanobacterium*, *Methanosarcina*, *Aspergillus*, *Penicillium*	Organic-rich substrates, methane, dust, moisture, geology and mining activity	Carbon degradation, methanogenesis and organic-matter mineralization	Dust aerosolization during coal cutting, drilling and transport	Microorganisms and microbial components may become airborne during mining activities; respiratory relevance depends on actual worker exposure and biological activity	[21,22]
Uranium mines	Radiation-tolerant and metal-tolerant bacteria and fungi	*Deinococcus*, *Ralstonia*, *Pseudomonas*, *Penicillium*, *Aspergillus*	Radioactive minerals, uranium, heavy metals, oxidative stress, nutrient limitation and pH	Radiation/metal tolerance, oxidative-stress adaptation, uranium transformation and immobilization	Inhalation of contaminated dust and contact with contaminated materials or water	Potential occupational exposure to microorganisms adapted to radiation and metal stress; direct evidence linking these microorganisms to occupational disease remains limited	[23,24,25]
Metal mines (Cu, Au, Fe)	Acidophilic bacteria, iron- and sulfur-oxidizing microorganisms, metal-tolerant fungi	*Acidithiobacillus*, *Leptospirillum*, *Sulfobacillus*, *Acidiphilium*, *Aspergillus*	Low pH, dissolved metals, sulfide minerals, redox conditions and mining activity	Iron/sulfur oxidation, bioleaching, acid generation and metal transformation	Dust, aerosols and contaminated mine-water systems	Potential exposure to aerosolized microorganisms and microbial components; environmental AMR findings require occupational validation	[26,27,28]
Acid mine drainage (AMD)	Acidophilic bacteria and archaea	*Ferroplasma*, *Leptospirillum*, *Acidithiobacillus*	Extremely low pH, metal-rich water and sulfur/iron substrates	Iron and sulfur oxidation, acid generation and metal mobilization	Water aerosols, splashing, drainage and contaminated sediments	Represents a potential environmental microbial reservoir; direct evidence for microbial disease among exposed workers is limited	[17]
Mine dust and bioaerosols	Bacteria and fungi	*Acinetobacter*, *Pseudomonas*, *Bacillus*, *Cladosporium*, *Aspergillus*	Particulate loading, ventilation, humidity and mining activity	Aerosolization, dispersal and persistence associated with mineral particles	Inhalation of dust-associated microorganisms, spores and microbial components generated during drilling, blasting and ore transport	Provides a plausible occupational inhalation pathway; respiratory, allergic and inflammatory effects require exposure–outcome evidence	[16,29]

Occupational interpretations in Table 1 are qualitative and were not generated using a validated evidence-grading instrument. They reflect the available evidence, including environmental detection, quantitative exposure measurements, occupational epidemiology, mechanistic evidence, and documented disease or outbreak investigations. These descriptors should not be interpreted as formal evidence-quality scores.

**Table 2:** Quantitative and qualitative evidence on microbial composition and bioaerosol exposure in mining environments.

Mining Environment/Exposure Source	Microbial Groups/Taxa	Reported Microbial Characteristics	Major Determinants of Exposure	Assessment Approach	Occupational Relevance	Reference
Coal mines—total and respirable dust	Gammaproteobacteria, Alphaproteobacteria, Betaproteobacteria; Firmicutes, Bacteroidetes and Actinobacteria	Gammaproteobacteria, Alphaproteobacteria and Betaproteobacteria together accounted for 72.5% of total bacterial sequences in coal-mine dust. Taxa containing potentially pathogenic bacterial species, including *Klebsiella*, *Burkholderia*, and *Aeromonas*, were detected in respirable dust.	Coal-derived organic substrates, excavation, ventilation, dust generation and mine geology	PacBio 16S rRNA sequencing; total and respirable dust sampling	Detection of potentially pathogenic taxa indicates possible biological exposure but does not establish species-level pathogenicity, strain-level virulence, viability, or occupational disease	[8]
Coal mines—underground microbial communities	Proteobacteria, Firmicutes, Actinobacteria, Bacteroidetes; methanogenic archaea including *Methanobacterium* and *Methanosarcina*	Methanogenic archaea are consistently detected in coal seams and underground coal environments; quantitative abundance varies substantially between sites and analytical methods.	Organic-rich substrates, methane, oxygen availability, moisture, geological characteristics and mining activity	Culture-independent community profiling/metagenomics	Provides an environmental reservoir of microorganisms that may become airborne during coal cutting, drilling and transport	[19,20,21,22,44]
Uranium mines	Radiation-resistant and metal-tolerant bacteria; *Deinococcus*, *Ralstonia*, *Pseudomonas*; metal-tolerant fungi including *Penicillium* and *Aspergillus*	Quantitative abundance estimates are heterogeneous and are generally reported as relative abundance or gene-level measurements rather than comparable airborne CFU/m^3^ values. Communities show adaptation to radiation, heavy metals and oxidative stress.	Radioactive minerals, uranium, heavy metals, oxidative stress, nutrient limitation and pH	Molecular community profiling and resistance-gene analyses	Potential exposure to metal/radiation-adapted microorganisms and environmental resistance determinants; direct microbial disease evidence remains limited	[23,24,25,45]
Metal mines (Cu, Au, Fe)	*Acidithiobacillus*, *Leptospirillum*, *Sulfobacillus*, *Acidiphilium*; metal-tolerant fungi	Microbial abundance varies with pH, metal concentration and mine-water chemistry; studies predominantly report community composition rather than directly comparable occupational aerosol concentrations.	Low pH, dissolved metals, sulfide minerals, redox conditions and mining activity	Culture-independent/community profiling and metagenomics	Potential exposure through contaminated dust, aerosols and mine water; environmental AMR implications require further occupational validation	[26,27,28,46]
Mine dust and bioaerosols	*Acinetobacter*, *Pseudomonas*, *Bacillus*, *Aspergillus*, *Penicillium*, *Cladosporium*	Microbial concentrations are highly variable between mines and sampling locations. Culture-based studies demonstrate viable microorganisms, while molecular studies detect microbial DNA and other microbial components in dust-associated aerosols.	Drilling, blasting, excavation, ore transport, ventilation, humidity and particulate loading	Culture-based and molecular bioaerosol assessment	Provides evidence of a plausible inhalation exposure pathway for microbial cells, spores, endotoxin and other microbial components	[15,16,29,30,31,32,33,34,35,36,37,38]
Landfill mining/mineralized refuse mining	Bacteria and fungi; *Arthrobacter*, *Bacillus*, *Pseudomonas*, *Arthrobotrys*, *Aspergillus*, *Penicillium*	Maximum bacterial aerosol concentration: 5968 ± 1608 CFU/m^3^ in the refuse-mining area; maximum fungal concentration: 1196 ± 370 CFU/m^3^ in the refuse-screening area. Bacterial aerosols were mainly 4.7–7.0 µm and fungal aerosols > 7.0 µm.	Excavation, screening, particulate matter, temperature, relative humidity and operational disturbance	Culture-based aerosol quantification, particle size analysis and metagenomic/pathogenicity assessment	Demonstrates that mining-related mechanical disturbance can generate substantial bioaerosol exposure; useful quantitative evidence for exposure assessment, although landfill mining differs from conventional mineral extraction	[16]
Mine water, drainage and biofilms	Bacteria, archaea and environmental ARGs; acidophilic and metal-tolerant taxa	High microbial diversity, including uncultured taxa and resistance determinants; quantitative results are generally reported as sequencing abundance or gene abundance rather than airborne CFU/m^3^.	pH, dissolved metals, water chemistry, redox conditions and biofilm formation	eDNA, 16S/ITS amplicon sequencing, shotgun metagenomics and resistance-gene analysis	Represents a potential environmental reservoir of microorganisms and ARGs; direct worker-health implications remain uncertain	[17,47,48,49,50,51]

Quantitative values are presented as reported in the underlying studies and were not pooled because culture-based counts, amplicon relative abundances, metagenomic read abundance, and gene-copy measurements represent different biological and analytical quantities. Detection of a taxon or resistance determinant does not establish viability, infectivity, strain-level pathogenicity, effective worker exposure, or disease causation.

**Table 3:** Microorganisms associated with respiratory exposure and reported or biologically plausible health outcomes in mining-related occupational environments.

Microorganism	Environmental Source	Reported or Biologically Plausible Health Outcome	Potential Exposure Pathway	Occupational Relevance	Evidence Type	References
*Aspergillus* spp.	Dust particles, ventilation systems, rock surfaces	Allergic bronchopulmonary aspergillosis; invasive pulmonary aspergillosis	Inhalation of airborne fungal spores during mining operations	Potential respiratory/allergic hazard; mining-specific causal evidence for aspergillosis remains limited	Environmental detection and general clinical/occupational evidence	[50]
*Histoplasma capsulatum*	Soil disturbed during excavation; bat/bird droppings in caves or mines	Histoplasmosis	Aerosolization of fungal spores during excavation, drilling or soil disturbance	Documented occupational disease risk in specific exposure settings	Epidemiological investigations and outbreak reports	[51]
*Streptomyces* spp.	Organic dust and soil residues	Hypersensitivity pneumonitis	Repeated inhalation of microbial/organic dust	Potential immune-mediated respiratory hazard	Occupational epidemiological evidence	[60]
*Pseudomonas* spp.	Environmental dust and mine-water systems	Opportunistic and respiratory infections	Inhalation of contaminated aerosols or dust	Potential exposure to opportunistic pathogens; direct mining-specific disease evidence limited	Environmental microbiological studies	[44]
Nontuberculous *Mycobacterium* spp.	Environmental water systems and dust particles	Pulmonary infections	Aerosol inhalation from contaminated water or dust	Potential occupational exposure; mining-specific causal evidence limited	Clinical and environmental studies	[45]

The presence of a microorganism in a mining environment should not be interpreted as evidence of occupational infection. For most taxa listed here, evidence supports environmental presence or biological plausibility rather than a demonstrated mining-acquired disease. The strongest mining-related occupational evidence in this table concerns specific exposure scenarios involving Histoplasma capsulatum. Allergic and hypersensitivity outcomes are classified as noninfectious respiratory outcomes.

**Table 4:** Conceptual framework for interpreting the strength and type of evidence on mining-associated microbiome risks.

Evidence Type	Operational Definition	Examples Relevant to Mining Microbiome Research	Interpretation	References
Direct occupational disease evidence	A defined occupational exposure is linked to a documented disease or health outcome through epidemiological investigation or an outbreak investigation	*Histoplasma capsulatum* exposure associated with histoplasmosis following exposure to contaminated soil, bat-associated material or mine/tunnel environments	Provides direct evidence of an occupational health association in the specific exposure setting studied	[55,59]
Epidemiological association	Worker exposure to microbial components, organic dust or fungal material is statistically or clinically associated with a health outcome, but causality is not definitively established	Exposure to microbial antigens, fungal material or organic dust associated with hypersensitivity pneumonitis or respiratory outcomes	Supports an exposure–health association but does not establish that a specific microorganism caused the outcome	[43,47,52,53,58]
Environmental detection	Microorganisms, microbial components or resistance determinants are detected in a mining environment without corresponding evidence of disease in exposed workers	Detection of *Aspergillus*, *Pseudomonas*, *Acinetobacter* and other taxa in mine dust, water or bioaerosols; detection of ARGs in environmental samples	Demonstrates environmental presence and potential exposure; does not establish viability, effective exposure, transmission or disease	[8,15,16,17,29,30,31,32,33,34,40,42,64,65]
Mechanistic evidence	Experimental, ecological or molecular findings support a biologically plausible mechanism, but the mechanism has not been adequately demonstrated in exposed mining workers	Microbe–mineral particle interactions, endotoxin-mediated inflammation, metal-associated selection/persistence of resistance determinants and potential horizontal gene transfer	Supports biological plausibility; occupational relevance requires direct exposure and health-outcome validation	[11,28,32,34,51,57,58]
Hypothesized pathway	A proposed exposure, transmission or health pathway lacks sufficient direct empirical evidence and requires targeted investigation	Possible transfer of mining-associated ARGs to human-associated microorganisms; possible additive or synergistic effects of microbial components and mineral dust	Should be presented as a research hypothesis rather than an established occupational risk	[44,47,50,51,60]

This is an author-developed conceptual framework for organizing heterogeneous evidence and is not a validated evidence-grading or risk-of-bias instrument. The categories describe the type of evidence available rather than assigning a numerical or hierarchical quality score. Environmental detection should not be equated with effective worker exposure, transmission, infection or disease.

**Table 5:** Comparison of reported microbial and bioaerosol findings across mining-related environments.

Setting	Environmental Compartment	Sampling/Analytical Approach	Main Finding	Quantitative Information Reported	Key Source of Heterogeneity	Occupational Interpretation	References
Underground hard-coal mine	Airborne/respirable dust	Culture-based microbial enumeration; community characterization	Substantial spatial variation in bacterial and fungal communities	Mesophilic bacterial concentrations approximately 1.65 × 10^3^ to >20 × 10^3^ CFU/m^3^, depending on sampling location	Sampling location and underground environmental conditions	Demonstrates measurable airborne microbial exposure; magnitude varies spatially	[16,29]
Coal-mine dust	Total and respirable dust	PacBio 16S rRNA sequencing	Proteobacterial classes represented a large proportion of bacterial sequences; potentially pathogenic taxa were detected	Gammaproteobacteria, Alphaproteobacteria and Betaproteobacteria collectively approximately 72.5% of bacterial sequences	Sequencing-based relative abundance cannot be directly compared with CFU/m^3^	Demonstrates microbial genetic diversity in respirable dust; does not establish viability or disease	[8]
Mineralized-refuse mining	Airborne aerosols	Culture-based aerosol enumeration and particle size assessment	Bacterial and fungal aerosols were measurable during mining-related mechanical disturbance	Bacteria: 5968 ± 1608 CFU/m^3^; fungi: 1196 ± 370 CFU/m^3^	Operational activity, particle size, temperature and relative humidity	Supports a plausible occupational bioaerosol exposure pathway	[16]
Mine water/drainage systems	Water, drainage and biofilms	eDNA, 16S/ITS sequencing, shotgun metagenomics and resistance-gene analysis	Diverse microbial communities and environmental resistance determinants detected	Generally reported as relative sequence abundance or gene abundance rather than airborne CFU/m^3^	Water chemistry, pH, metals, redox conditions and biofilm structure	Indicates an environmental reservoir; worker-health relevance remains uncertain	[17,18,62,64,65]
Metal/acid mine environments	Mine water, sediments, surfaces and associated aerosols	Culture-independent community profiling and metagenomics	Acidophilic and metal-tolerant microorganisms associated with low-pH and metal-rich environments	Quantitative results vary substantially between studies	pH, metal concentrations, mineralogy and sampling compartment	Demonstrates ecological adaptation; occupational exposure requires direct measurement	[26,27,28,46]

Reported quantitative measurements are study-specific and should not be interpreted as pooled estimates. Differences in sampling volume, particle size fraction, culture conditions, sequencing platform, molecular target, bioinformatic pipeline and reporting units limit direct quantitative comparison.

## Data Availability

No new data were created or analyzed in this study. Data sharing is not applicable to this article.

## References

[1] Yu H., Gao Y., Zhou R. (2020). Oxidative Stress from Exposure to the Underground Space Environment. Front. Public Health.

[2] Gbondo D., Zhao Y., Pham M., Rumchev K. (2024). Trends in Exposure to Respirable Dust and Respirable Crystalline Silica Among Lithium Mine Workers in Western Australia. Saf. Health Work.

[3] Hua J.T., Farah W.H., Rose C.S., Kellermeyer L., Zell-Baran L.M. (2026). Silicosis and silica dust sampling in mineral mining across 30 countries: A systematic review and meta-analysis. BMC Public Health.

[4] Thavamani P., Samkumar R.A., Satheesh V., Subashchandrabose S.R., Ramadass K., Naidu R., Venkateswarlu K., Megharaj M. (2017). Microbes from mined sites: Harnessing their potential for reclamation of derelict mine sites. Environ. Pollut..

[5] Mulikita S., Ponraj M., Mutondo M., Maseka K. (2026). Effects of mining pollution and climate change on microbial communities and human health. Pedosphere.

[6] Rastmanesh A., Boruah J.S., Lee M.S., Park S. (2024). On-Site Bioaerosol Sampling and Airborne Microorganism Detection Technologies. Biosensors.

[7] Iqbal M.A., Siddiqua S.A., Faruk M.O., Md Towfiqul Islam A.R., Salam M.A. (2024). Systematic review and meta-analysis of the potential threats to respiratory health from microbial Bioaerosol exposures. Environ. Pollut..

[8] Xue S., Liu X., Li Y., Liu B., Tu Q., Li C. (2022). Pathogenic Bacterial Communities of Dust in a Coal Mine. Front. Environ. Sci..

[9] Chen J., Sun L., Liu X., Yu Q., Qin K., Cao X., Gu J. (2022). Metagenomic Assessment of the Pathogenic Risk of Microorganisms in Sputum of Postoperative Patients with Pulmonary Infection. Front. Cell. Infect. Microbiol..

[10] Xiao Y., He J.-W., Cao Y., Wang Z.-P., Deng J., Shu C.-M. (2026). Migration patterns of underground mine dust in the past two decades based upon the methods of bibliometric and visual analyses. J. Loss Prev. Process Ind..

[11] Wan G.H., Li C.S. (1999). Indoor endotoxin and glucan in association with airway inflammation and systemic symptoms. Arch. Environ. Health.

[12] Mohammadi A., Soleimani A., Abdolahnejad A., Ahmed M., Akther T., Nemati-Mansour S., Raeghi S., Rashedi G.H., Miri M. (2022). SARS-CoV-2 detection in hospital indoor environments, NW Iran. Atmos. Pollut. Res..

[13] Zou H.Y., Gao F.Z., He L.Y., Zhang M., Liu Y.S., Qi J., Ying G.G. (2025). Prevalence of antibiotic resistance genes in mining-impacted farmland environments. Ecotoxicol. Environ. Saf..

[14] Jones S., Santini J.M. (2023). Mechanisms of bioleaching: Iron and sulfur oxidation by acidophilic microorganisms. Essays Biochem..

[15] Li W., Feng Q., Southam G., Jin T., Li Z. (2023). Changes in microbial community structure during the biooxidation of iron and inorganic/organic sulfur provide prediction of acid mine drainage from coal spoil. Sci. Total Environ..

[16] Nóbrega P.D.A., Ferreira B.L., Sá L.S., Braga F.S., Bezerra R.M., Ferreira I.M. (2024). Isolation and Identification of Iron, Chromium, and Manganese Tolerant Filamentous Fungi from Mining Soil. Curr. Biotechnol..

[17] Wang Y.-N., Wang Q., Li Y., Wang B., Wang H., Sun Y., Wu J., Li W., Bian R., Chen L. (2024). Insights into bioaerosol contamination in the process of mineralized refuse mining: Microbial aerosolization behavior and potential pathogenicity. J. Hazard. Mater..

[18] Chen L.-X., Hu M., Huang L.-N., Hua Z.-S., Kuang J.-L., Li S.-J., Shu W.-S. (2014). Comparative metagenomic and metatranscriptomic analyses of microbial communities in acid mine drainage. ISME J..

[19] Dolkar P., Modeel S., Siwach S., Yadav P., Chaurasia M., Themchurin L., Yadav A., Atri A., Kapoor S., Negi R.K., Gadd G.M., Sariaslani S. (2025). Metagenomic analysis of metal(loid)s resistance genes and its environmental applications. Advances in Applied Microbiology.

[20] Wei M., Yu Z., Jiang Z., Zhang H. (2014). Microbial diversity and biogenic methane potential of a thermogenic-gas coal mine. Int. J. Coal Geol..

[21] Wei X., Chen H., Zhu F., Li J. (2024). Microbial community structure in an uranium-rich acid mine drainage site: Implication for the biogeochemical release of uranium. Front. Microbiol..

[22] Campbell B.C., Greenfield P., Gong S., Barnhart E.P., Midgley D.J., Paulsen I.T., George S.C. (2022). Methanogenic archaea in subsurface coal seams are biogeographically distinct: An analysis of metagenomically-derived mcrA sequences. Environ. Microbiol..

[23] Fuentes-Alburquenque S., Olivencia Suez V., Aguilera O., Águila B., Rojas Araya L., Mandakovic D. (2024). A Highly Homogeneous Airborne Fungal Community around a Copper Open Pit Mine Reveals the Poor Contribution Made by the Local Aerosolization of Particles. Microorganisms.

[24] Wufuer R., Waheed A., Pei L., Duo J., Wang S., Li W., Yang F., Chimezie E.C. (2025). Microbial interactions with uranium: Mechanisms, ecosystem impacts, and bioremediation strategies. Environ. Technol. Innov..

[25] Chen H., Sheng Y., Wang S., Chen Y., Qiao Z., Guo H., Dong H. (2025). Uranium contamination mediating soil and ore microbial community assembly at four mining sites, South China. Front. Microbiol..

[26] Coelho E., Reis T.A., Cotrim M., Mullan T.K., Corrêa B. (2020). Resistant fungi isolated from contaminated uranium mine in Brazil shows a high capacity to uptake uranium from water. Chemosphere.

[27] Hetz S.A., Schippers A. (2025). Aerobic Bioleaching of Six Brazilian Laterite Ores with *Acidithiobacillus thiooxidans*, *Sulfobacillus* species and Archaea at Various Conditions. J. Sustain. Metall..

[28] Baker B.J., Banfield J.F. (2003). Microbial communities in acid mine drainage. FEMS Microbiol. Ecol..

[29] Newsome L., Falagán C. (2021). The Microbiology of Metal Mine Waste: Bioremediation Applications and Implications for Planetary Health. GeoHealth.

[30] Cheluszka P., Paśmionka I.B., Gospodarek J., Vieira F.M.C. (2023). The spread of microbiota in the air of an underground hard coal mine—A case study. Build. Environ..

[31] Zhang L., Xu Z., Sun Y., Gao Y., Zhu L. (2022). Coal Mining Activities Driving the Changes in Microbial Community and Hydrochemical Characteristics of Underground Mine Water. Int. J. Environ. Res. Public Health.

[32] Jasim S.A., Mohammadi M.J., Patra I., Jalil A.T., Taherian M., Abdullaeva U.Y., Sharma S., Ekrami H.A., Mousavion K., Alborzi M. (2024). The effect of microorganisms (bacteria and fungi) in dust storm on human health. Rev. Environ. Health.

[33] Poole J.A., Zamora-Sifuentes J.L., De Las Vecillas L., Quirce S. (2024). Respiratory Diseases Associated with Organic Dust Exposure. J. Allergy Clin. Immunol. Pract..

[34] Yang J., Kim J.S., Jeon H.W., Lee J., Seo J.H. (2025). Integrated culture-based and metagenomic profiling of airborne and surface-deposited bacterial communities in residential environments. Environ. Pollut..

[35] Hu W., Murata K., Fan C., Huang S., Matsusaki H., Fu P., Zhang D. (2020). Abundance and viability of particle-attached and free-floating bacteria in dusty and nondusty air. Biogeosciences.

[36] Liu F., Nie W., Peng H., Niu W., Li X. (2026). Mechanism of multi-scale supersonic droplet atomization for dust suppression in tunnel excavation and coal mining. Tunn. Undergr. Space Technol..

[37] Nie W., Liu F., Niu W., Tian Q., Li R. (2026). A multiscale study on the molecular mechanisms of surfactants that enhance the wettability of coal dust and the efficiency of droplet coagulation. J. Clean. Prod..

[38] Niu W., Nie W., Yang W., Tian Q. (2026). Mechanism and design strategy via surfactant hydrophobic tail engineering for enhanced dust control in underground spaces. Tunn. Undergr. Space Technol..

[39] Lee S., Jo A.R., Kim Y., Lee W., Ma X. (2025). Association between occupational and environmental dust exposure and autoimmune diseases: A systematic review and meta-analysis. J. Autoimmun..

[40] Huang R., Chen W., Tao Y., Yuan S., Geng F., Li S. (2024). Influence of environmental parameters on workers’ dust inhalation in underground mines. Environ. Sci. Pollut. Res. Int..

[41] Al-Shaarani A., Pecoraro L. (2024). A review of pathogenic airborne fungi and bacteria: Unveiling occurrence, sources, and profound human health implication. Front. Microbiol..

[42] Skubacz K., Hildebrandt R., Zgórska A., Dyduch Z., Samolej K., Smolinski A. (2021). Transport of Aerosols in Underground Mine Workings in Terms of SARS-CoV-2 Virus Threat. Molecules.

[43] Best Mackenzie B., Kazemi Motlagh Z., McLemore Virginia T., Jones Daniel S. (2025). Historic mine waste contains diverse microbial communities that reflect waste type and geochemistry. Appl. Environ. Microbiol..

[44] LeChevallier M.W., Prosser T., Stevens M. (2024). Opportunistic Pathogens in Drinking Water Distribution Systems—A Review. Microorganisms.

[45] Falkinham J.O. (2015). Environmental sources of nontuberculous mycobacteria. Clin. Chest Med..

[46] Daae H.L., Graff P., Foss O.A.H., Kofoed V.C., Afanou A.K., Frederiksen M.W., Madsen A.M. (2024). A cross-sectional study on occupational exposure to microorganisms, endotoxin, hydrogen sulfide, and dust during work at drilling waste treatment plants. Ann. Work Expo. Health.

[47] Liebers V., Brüning T., Raulf M. (2020). Occupational endotoxin exposure and health effects. Arch. Toxicol..

[48] Kamanzi C., Becker M., Jacobs M., Konečný P., Von Holdt J., Broadhurst J. (2023). The impact of coal mine dust characteristics on pathways to respiratory harm: Investigating the pneumoconiotic potency of coals. Environ. Geochem. Health.

[49] Armstrong P.A., Beard J.D., Bonilla L., Arboleda N., Lindsley M.D., Chae S.R., Castillo D., Nuñez R., Chiller T., de Perio M.A. (2018). Outbreak of Severe Histoplasmosis Among Tunnel Workers-Dominican Republic, 2015. Clin. Infect. Dis..

[50] Chantelot L., Sitterle E., Poiree S., Jullien V., Danion F., Serris A. (2026). Cerebral Aspergillosis: Diagnostic Challenges, Therapeutic Strategies, and Future Research. CNS Drugs.

[51] Muñoz-Rosa M., Rhyman N., Tobón-Orozco Á.M. (2025). Histoplasmosis: An endemic mycosis. Overview of epidemiology, clinical features, diagnosis, and treatment. Infectio.

[52] Zhang X., Igor B., Elena D., Olga R., Glazachev O. (2024). Prevalence of occupational hypersensitivity pneumonitis: A systematic review and meta-analysis. Int. J. Environ. Health Res..

[53] Andrews K., Ghosh M.C., Schwingshackl A., Rapalo G., Luellen C., Waters C.M., Fitzpatrick E.A. (2016). Chronic hypersensitivity pneumonitis caused by Saccharopolyspora rectivirgula is not associated with a switch to a Th2 response. Am. J. Physiol. Lung Cell. Mol. Physiol..

[54] Gomes M.L., Morais A., Cavaleiro Rufo J. (2021). The association between fungi exposure and hypersensitivity pneumonitis: A systematic review. Porto Biomed. J..

[55] Elkhapery A., Fatima M., Soubani A.O. (2025). Emerging Risk Factors for Invasive Pulmonary Aspergillosis: A Narrative Review. J. Fungi.

[56] de Perio M.A., Benedict K., Williams S.L., Niemeier-Walsh C., Green B.J., Coffey C., Di Giuseppe M., Toda M., Park J.-H., Bailey R.L. (2021). Occupational Histoplasmosis: Epidemiology and Prevention Measures. J. Fungi.

[57] Howard-Jones A.R., Sparks R., Pham D., Halliday C., Beardsley J., Chen S.C.A. (2022). Pulmonary Cryptococcosis. J. Fungi.

[58] Abbas M., Khan M.T., Iqbal Z., Ali A., Eddine B.T., Yousaf N., Wei D. (2024). Sources, transmission and hospital-associated outbreaks of nontuberculous mycobacteria: A review. Future Microbiol..

[59] García-Martín J.M., López J.D.S., Lizana-Ciudad D., Fernández-Soto P., Muro A. (2025). Molecular detection of histoplasma in bat-inhabited tunnels of Camino de Hierro Tourist Route, Spain. Emerg. Infect. Dis..

[60] Iversen I.B., Vestergaard J.M., Basinas I., Ohlander J., Peters S., Bendstrup E., Bonde J.P.E., Schlünssen V., Rasmussen F., Stokholm Z.A. (2024). Risk of hypersensitivity pneumonitis and other interstitial lung diseases following organic dust exposure. Thorax.

[61] Michaelis C., Grohmann E. (2023). Horizontal Gene Transfer of Antibiotic Resistance Genes in Biofilms. Antibiotics.

[62] Zhang J., Lei H., Huang J., Wong J.W.C., Li B. (2025). Co-occurrence and co-expression of antibiotic, biocide, and metal resistance genes with mobile genetic elements in microbial communities subjected to long-term antibiotic pressure: Novel insights from metagenomics and metatranscriptomics. J. Hazard. Mater..

[63] Han X., Liu H., Bai X., Li D., Wang T., Zhong H., Yao Y., Sun J. (2025). Insights into antibiotic resistomes from metagenome-assembled genomes and gene catalogs of soil microbiota across environments. PeerJ.

[64] Hagarová L., Kupka D. (2025). Insights into the microbiome of mine drainage from the Mária mine in Rožňava, Slovakia: A metagenomic approach. Front. Microbiol..

[65] Fulke A.B., Sharma N., Nadekar J. (2025). Darkness to Discovery: A Comprehensive Mini-Review on Culturable and Non-Culturable Microbial Diversity from Deep Sea. Microb. Ecol..

[66] Wani A.K., Akhtar N., Naqash N., Rahayu F., Djajadi D., Chopra C., Singh R., Mulla S.I., Sher F., Américo-Pinheiro J.H.P. (2023). Discovering untapped microbial communities through metagenomics for microplastic remediation: Recent advances, challenges, and way forward. Environ. Sci. Pollut. Res..

[67] Laudadio I., Fulci V., Palone F., Stronati L., Cucchiara S., Carissimi C. (2018). Quantitative assessment of shotgun metagenomics and 16S rDNA amplicon sequencing in the study of human gut microbiome. OMICS J. Integr. Biol..

[68] Rishan S.T., Kline R.J., Rahman M.S. (2025). Environmental DNA (eDNA) collection techniques across diverse ecosystems: A mini-review of promising new tools for eDNA metabarcoding. Environ. Sci. Pollut. Res. Int..

[69] Child H.T., Wierzbicki L., Joslin G.R., Roper K., Haxhiraj Q., Tennant R.K. (2025). Automated environmental metagenomics using Oxford nanopore sequencing. BMC Genom..

[70] Hudu S.A., Morad E.A., Alhazimi G.M., Jimoh A.O. (2026). Heavy Metal Pollution in Mining Ecosystems: An Emerging Driver of Environmental Resistomes and Antimicrobial Resistance. Environments.

[71] Tian L., Lu J.-N., Zhang Y., Zhang Q., Jiang G., Yin Y., Li L., Fei Y.-H., Yang Y., Ruan Z. (2026). Overlooked dissemination risk of resistomes in mining soil environments. J. Hazard. Mater..

